# Development of the Food Boost Challenge: A Participatory Action Research Approach to Enhance Vegetable and Fruit Consumption among Adolescents

**DOI:** 10.3390/nu15234921

**Published:** 2023-11-25

**Authors:** Machteld van Lieshout, Wendy Scholtes-Bos, Judith M. van der Horst-Graat, Puck van Holsteijn, Sanne I. de Vries

**Affiliations:** 1 Research Group Healthy Lifestyle in a Supporting Environment, Centre of Expertise Health Innovation, The Hague University of Applied Sciences, 2521 EN The Hague, The Netherlands; w.scholtes-bos@kftrw.nl (W.S.-B.); s.i.devries@hhs.nl (S.I.d.V.); 2 Department of Nutrition & Dietetics, Faculty of Health, Nutrition & Sports, The Hague University of Applied Sciences, 2521 EN The Hague, The Netherlands; 3 Medical Delta Living Lab VIT for Life, Medical Delta, 2629 JH Delft, The Netherlands; 4 Foodvalley NL, 6708 WH Wageningen, The Netherlands; judith.vanderhorst@foodvalley.nl; 5 HortiHeroes, 2672 ZX Naaldwijk, The Netherlands; puck.van.holsteijn@hortiheroes.com; 6 Department of Public Health and Primary Care, Health Campus The Hague, Leiden University Medical Center, 2511 DP The Hague, The Netherlands

**Keywords:** food system, peer research, cocreation, lifestyle, healthy diet, healthy eating behavior, noncommunicable diseases, prevention, adolescents, school students

## Abstract

Prevention of non-communicable diseases through, among other factors, increasing vegetables and fruit (V&F) intake is a cost-effective strategy for risk reduction but requires behavioral change. Such changes in adolescents benefit from their active involvement. The Food Boost Challenge (FBC) was developed using a participatory action research approach to enhance healthy eating behaviors, namely V&F products among adolescents. The FBC is an innovation process, involving adolescents, (peer) researchers, and food system partners, like non-governmental and commercial organizations. In 2021–2022, 34 partners provided both cash and in-kind contributions to join the FBC community. Phase 1 involved 200 students identifying barriers and drivers for consumption of F&V products among 1000 pre-vocational adolescents, aged 12–20 years. In phase 2, student teams submitted innovative ideas, resulting in 25 concepts fitting into ≥1 of 4 routes: (I) innovative technology for a healthy diet, (II) new food products/concepts for adolescents, (III) hotspots improving the F&V product experience, and (IV) new routes to market. In phase 3, consortia of adolescents, students, and partners were formed to develop 10 selected concepts into prototypes, and phase 4 offered teams a national platform. Results show that the FBC resonates with all stakeholders, generating valuable insights to increase F&V intake. Prototypes in all four routes have been developed. Additionally, other regions in the Netherlands have adopted the FBC approach. Overall, the FBC is an approach that transforms ideas into actionable measures and shows potential to be adapted to promote various healthy eating behaviors among school students.

## 1. Introduction

Noncommunicable diseases (NCDs), such as cardiovascular diseases, cancers, chronic respiratory diseases, and diabetes, were, in the pre-COVID years, responsible for over 70% of deaths globally [1]. In high-income countries, NCDs are overrepresented in people with lower socioeconomic positions (i.e., those with lower education, occupational class, or income [2]), thereby limiting health equality. In 2016, 90% of deaths in the Netherlands, a country in Northern Europe, were attributed to NCDs. About 11% of deaths are premature, between the age of 30 and 70 years old, and are caused by NCDs [3]. Although both NCD prevention and control are essential response strategies for countries at all income levels, NCD prevention is more cost-effective than control [4].

With respect to NCD prevention, an unhealthy diet is one of the four main modifiable behavioral risk factors underlying NCDs (the others being tobacco use, physical inactivity, and harmful use of alcohol) [1]. The 2015 Global Burden of Disease study calculated that diets (1) high in sodium, (2) low in vegetables, (3) low in fruit, (4) low in whole grains, (5) low in nuts and seeds, and (6) low in seafood omega-3, each accounted for more than 1% of global disability-adjusted life years (DALYs). This stresses the importance of promoting both the reduction in sodium intake and the increase in intake of vegetables, fruit, whole grains, nuts, seeds, and seafood omega-3 through interventions, such as education, subsidies, and other evidence-based strategies [5]. This is fully in line with the World Health Organization’s (WHO’s) 2013–2020 global action plan for the prevention and control of noncommunicable diseases aimed at “reducing the preventable and avoidable burden of morbidity, mortality and disability due to noncommunicable diseases by means of multisectoral collaboration and cooperation at national, regional and global levels, so that populations reach the highest attainable standards of health and productivity at every age and those diseases are no longer a barrier to well-being or socio-economic development” [6]. Their overarching principles for achieving this goal include, among others, the following: (1) life course approach; (2) equity-based approach; (3) multisectoral action; (4) empowerment of people and communities; (5) evidence-based strategies.

When zooming in on these principles, the following gaps and opportunities are identified. Firstly, NCD prevention often focuses on either the first 1000 days or on the general adult population, while adolescents have been overlooked [7]. This neglect has been postulated to be the result of a lack of data about this target group and of evidence of what policies, strategies, and interventions work in this target group [8]. However, adolescence is a unique window of opportunity for the development of autonomous health promoting behavior and for limiting behavioral risk factors. Their cognitive function is larger than during childhood, the period with even larger brain plasticity, and their ability for change might be even larger than during adulthood [9]. Crone and Dahl [10] define adolescence as “a phase of development characterized by flexible adaptation to a rapidly changing social landscape marked by changes from dependency to autonomy and individuality”. Not only their social landscape, but also their food environment rapidly changes [7]. In 2019 in the Netherlands, 67% of secondary schools had at least one food outlet within five minutes walking. Schools in low-income neighborhoods have a higher chance of having at least one nearby food outlet than schools in high-income neighborhoods [11].

Targeting adolescents in pre-vocational schools aligns with the equity-based approach, the above-mentioned second principle, as these students often reside in and attend schools within low-income neighborhoods. They deserve heightened attention. As a result of intergenerational effects, they are more susceptible to poorer health from conception through childhood [12]. Investment in their health would also be a chance to break this intergenerational cycle and propel them, and eventually their offset, into a better position in life.

Changing the food system of adolescents requires a multisectoral approach, the third principle, as also proposed in WHO’s Global Accelerated Action for the Health of Adolescents (AA-HA!) [13]. A multisectoral approach requires commitment of all parties, including the target group. Adolescents themselves can be peer researchers or citizen scientists for understanding the “present”, identifying levers for “change”, and for cocreating their “future”. This approach has been successfully applied to address upstream NCD risk factors in urban low- and middle-income contexts [14].

Giving adolescents a voice would not only empower them, the fourth principle, but their brain development also demands such an approach. Adult feedback is far less effective for changing their behavior compared to peer feedback. Also, under the right circumstances—such as when they are highly motivated—their problem-solving skills may well be better than those of adults as they have a large capacity to diverge during a creative process [10]. Do not tell them what to do, but rather ask them for solutions.

We applied this approach for the design of a so-called social innovation—the Food Boost Challenge. The Dutch Advisory Council for Science and Technology Policy defines social innovation as “new solutions that simultaneously meet a societal need and introduce or improve capacities and relationships and a better use of resources”. They state that “social innovations are good for society and increase its capacity for action” [15]. Our study sought to address a critical gap in evidence-based strategies focusing on enhancing healthy eating behaviors among pre-vocational adolescents, aged 12–20 years old. The lack of such strategies, especially tailored for this specific demographic, prompted the development of the Food Boost Challenge. For this first edition, we selected two of the above-mentioned six dietary factors which favorably affect DALY and contribute to the prevention of NCDs, namely promoting the intake of vegetables and fruit. Very few people aged 1–79 years old in the Netherlands consume the recommended daily minimum of 250 g of vegetables and 200 g of fruit (i.e., 6% and 15%, respectively). In the last national food consumption survey, adolescents, aged 14–18 years old, consumed, on average, about 100 g of vegetables and 100 g of fruit per day, with 1% reaching the recommendation for vegetables and 7–10% reaching the recommendation for fruit (boys–girls, respectively) [16]. As such, there is sufficient room for improvement in the intake of vegetable and fruit products among adolescents. To our knowledge, there is no evidence-based strategy—the fifth WHO principle—in the Netherlands nor elsewhere that focuses on stimulating the intake of vegetable and fruit products among pre-vocational adolescents (12–20 years old). In this paper, we describe the design of the social innovation called the Food Boost Challenge (FBC) that has been developed for this purpose. For each phase of the FBC, justification for methodological choices will be provided in the Materials and Methods section. Subsequently, we present the initial results of each phase of the FBC. Finally, in the discussion, we share key drivers for its success, identify areas of improvement, and reflect on the significance of the results for enhancing the intake of vegetable and fruit among adolescents.

## 2. Materials and Methods

**Figure 1:**
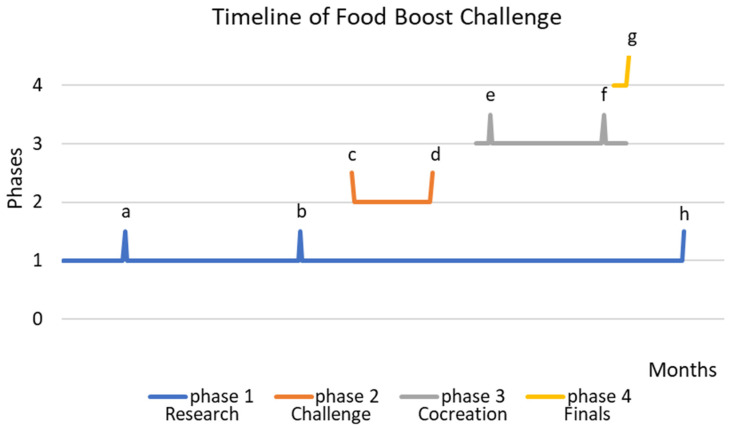
Timeline, in days, of phases and events (indicated as spikes) of the Food Boost Challenge. See Table 1 and text for a full description of phases and events. In brief, spike a and b represent partner meetings for kick-off and knowledge circulation, respectively, until spike h; spike c and d represent the start and end of the challenge phase during which student teams could submit concepts; spike e represent the matchmaking and cocreation day during which consortia of adolescents, student teams and partners were formed and validation of prototypes was started; spike f represents pitch training for students teams; and spike g represents the national finals.

The design of the Food Boost Challenge is based on participatory action research. In 2019, Chevalier and Buckles stated that participatory action research is “often intentionally defined only broadly in order to promote pluralism and creativity in the art of discovering the world and making it better at the same time” [17]. However, all definitions agree that participatory action research is based on a combination of three essential pillars, namely direct engagement of participants, transformative action towards empowering people for societal issues, and advancement of knowledge through research [17,18]. The aim of our study, developing an approach which enhances consumption of vegetables and fruit among adolescents, guided us toward using qualitative participatory action research as methodological approach. This approach enables the following: (1) direct engagement with the target group, which is especially relevant in adolescents as they are becoming increasingly more independent in their nutritional behavior; (2) transforming ideas into actionable measures which impact a societal issue, i.e., healthy eating behavior; (3) generating knowledge and insights answering the question “what do adolescents need for enhancing their consumption of vegetables and fruit”. In Figure 1, a complete timeline of phases and events (Spikes a–h) of the Food Boost Challenge is provided. In February 2021, the founding partners of the Food Boost Challenge, namely Foodvalley NL, HortiHeroes, and The Hague University of Applied Sciences along with Medical Delta Living Lab VIT for Life, conceived the Food Boost Challenge for stimulating the intake of vegetable and fruit products among pre-vocational adolescents. Herewith, the developmental phase of the Food Boost Challenge was initiated. A swift start was made, recruiting partners throughout the whole food system covering the adolescents’ ecosystem, aspiring for at least 30 partners. The collaboration with partners throughout the food system was deemed important to not only generate knowledge, but also to achieve change in real life. Partners were asked to sign a letter of commitment confirming both a pro rata cash and an in-kind contribution to the FBC. Cash contributions would be used for catering, workshop material, and photo and video reports of meetings, etc. In-kind contributions would consist of providing space for meetings, catering, sharing of knowledge and expertise, goodie bags, etc. Additional funding was being sought through grant applications. After the initial 150 days of partner recruitment, it was decided to go ahead with the challenge even if no additional grant funding could be acquired. This decision was merely based on the immediate buy-in of a wide array of partners supporting both the goal and approach of the Food Boost Challenge. This coincided with the official launch of the Food Boost Challenge in July 2021.

In August 2021, the research phase, phase 1 of the Food Boost Challenge, started (until spike h, Figure 1). The aim of this phase was to identify barriers and drivers of adolescents aged 12–20 years old, for changes in the consumption of vegetable and fruit products. General research questions were formulated by the research team and partners were offered the opportunity to add specific research questions. All questions were related to the above-mentioned main question “what do adolescents need for enhancing their consumption of vegetables and fruit?” Throughout the entire research phase, students, scholars, and their teachers at research and pre-vocational schools were invited to participate in a variety of intra-curricular projects, preferably using innovative methodologies. There was ample room for engaging as long as their participation contributed towards the above-mentioned aim of this research phase (i.e., stimulating the consumption of vegetable and fruit products). In order to enable students to meet their varying intra-curricular demands, no further requirements were set for applying specific research methods. Therefore, students were free to choose qualitative or quantitative research methods. We aspired to involve a minimum of 200 students and scholars in this phase. The kick-off meeting for partners was held towards the end of September 2021 (see Figure 1, spike a). The goal of this meeting was for partners and the core team to meet and connect, and for partners to be briefed about what to expect in each phase of the Food Boost Challenge. Spike b (Figure 1) refers to the partner meeting in December 2021, during which the initial results of the research phase were shared. This circulation of knowledge, gathered in the research phase, is an important characteristic of participatory action research [18].

Early in 2022, during a 6-week-long period (see Figure 1, spike c for the start and spike d for the end), students, 16–28 years old, were challenged to develop innovative ideas into concepts that would increase consumption of vegetable and fruit products among 12–20-year-olds. The minimum age of 16 years was a pragmatic choice guided by the possibilities for students to travel to evening sessions without difficulties. Students were reached via the core team’s network, partners, Dutch universities, social media, radio interviews, etc. Concepts had to fit into at least one of four routes: (I) innovative technology to stimulate a healthy diet; (II) new food products/concepts targeting adolescents; (III) hotspots, i.e., physical places and/or events, influencing and improving the experience of F&V products; (IV) new routes to markets, e.g., new channels and/or ways of presenting products. A jury, consisting of members of the core team, reviewed all applications submitted before the deadline (see Figure 1, spike d), looking at innovativeness, relevance and potential impact, team composition, and ambition.

The most promising student teams entered phase 3 of the Food Boost Challenge: the matchmaking and cocreation phase. We aspired to collect at least 50 innovative ideas. of which 15 would be selected for participation in phase 3. Shortly before the start of phase 3 (see Figure 1, spike e) partner recruitment stopped, since new partners would be unable to be matched if entering beyond this moment. See the project website for a complete list of partners [19]. Phase 3 started with an event at which knowledge and insights from the research phase were shared with all participants and partners. In line with the pillars of participatory action research, this is an empowering step [18]. In mid-March 2022 (see Figure 1, spike e), all student teams, partners, and representatives of the target group met during four rounds of speed dates, after which they were matched based on personal and professional preferences. These newly formed consortia of a student team and 1–2 partners and representatives of the target group subsequently received a professional cocreation training to kickstart their prototyping phase. This approach was chosen as it contributes both to empowering all involved and to transforming ideas into action, both of which are important elements of participatory action research [17,18]. Throughout this phase, these consortia continued to develop and validate their prototypes. Phase 3 also ended with an event. In mid-May 2022 (see Figure 1, spike f), all student teams received a professional pitch training after which their pitch was video-taped. This training not only served as professional development for participants, but also enabled them to further reflect on the essence of their prototypes. This reflection is an important element of participatory action research. These pitches were used during a one-week-period of public voting at the start of phase 4 of the Food Boost Challenge.

Phase 4, which intended to create a national buzz and a boost for a healthy diet among adolescents, ended with the national finals (see Figure 1, spike g), a one-day event, during which consortia and partners had a national stage at which prototypes were pitched by the student teams and experienced by the jury and audience. Research findings were also shared, and all people present networked. Four prizes were awarded to the student teams during the finals. Winners were identified by public voting, a jury process. a partner selection process, and an encouragement award. The expert jury looked at quality of video and live pitch, the validation of prototype, potential for impact, and team credentials. We aspired to have a minimum audience of 100 people during the national finals.

## 3. Results

In Table 1, the design of the Food Boost Challenge is summarized, including numbers reached. These are important parameters for evaluating the adequacy of the structure of the Food Boost Challenge. Most remarkable was the large buy-in for the Food Boost Challenge of partners, teachers, students, and scholars. This commitment enabled the generation of sufficient funding and knowledge sharing, intra-curricular exposure to the importance of a healthy diet in general and vegetable and fruit products in particular for >2000 scholars, and active participation in a quadruple helix project for >200 students. In Table 2, all pre-set goals are compared with actual achievements. As can be seen, five of the seven goals were reached. For one goal, the goal was deliberately lowered. Because 25 concepts were submitted rather than 50, only 10—not 15—of those were chosen to enter the matchmaking and cocreation phase. Table 3 lists the prototypes pitched at the finals. Apart from the numbers of partners, students, teams, adolescents participating, the outcome of this challenge was hard to quantify; however, we do consider the FBC approach to be viable because (1) most participants enjoyed participation, (2) the FBC approach was adopted in another region in the Netherlands (the Food Boost Challenge Limburg), (3) advanced plans, including a submitted grant application, for future editions of the FBC in the Netherlands and abroad were developed, and (4) several partners expressed the desire to continue participating in future editions of the FBC.

In phase 1, students were chosen from eight different departments of The Hague University of Applied Sciences, namely Nutrition and Dietetics, User Experience, Business Administration, Process and Food Technology, Applied Mathematics, Communication, ICT and HRM. They used a variety of research methods, such as spy-on-the-wall observations, focus groups and informal interviews in school canteens, tastings, questionnaires, and in-class assignments and activities. The in-class assignments and activities involved mind-maps; interactive games, such as “cross the line”, the “sticky notes game”, a “would you rather—choice game”, and an “association game”, as well as classroom quizzes using digital tools. These digital tools were interactive versions of questionnaires, such as the Kahoot quiz and Socrative.

In phase 2, no information about the research results of phase 1 could be shared with participants, since they only became known when entering their concept.

In phase 3, students were chosen from 10 different universities throughout The Netherlands. Almost half of the teams attended a so-called “*green university*”, with its origin in agricultural research and education. For all students, this was an extra-curricular activity.

In phase 4, the prizes were awarded to the following teams (see Table 3). Team Power Tower won the public vote. Team Veggie Smooth won the jury price. Team Veggie Smooth was also selected to pitch their prototype on the overseas location of their partner App Harvest for their American investors. Team Good Food Mood App won the encouragement award.

## 4. Discussion

In this paper, we described the design of a social innovation called the Food Boost Challenge that has been developed for increasing the intake of vegetable and fruit products in adolescents. Overall, we conclude that the proposed approach for the Food Boost Challenge could transform ideas into actionable measures and shows potential to be adapted to promote various healthy eating behaviors among school students. In addition to the initial results, we also identified key drivers for its success. Results indicate this quadruple helix innovation hits the right notes with all involved. The Food Boost Challenge generated useful insights into what adolescents need for increasing their intake of vegetable and fruit products. Not only have prototypes been developed in all four routes [20] (see Table 3), but some of them have already reached the implementation stage. In addition, the Food Boost Challenge is a sustainable model as it will be repeated and adopted in other regions and settings in The Netherlands and possibly abroad.

For future editions of the Food Boost Challenge, we encountered areas of improvement, larger potential, and research needs. These are discussed using the main learnings 1–6 in Table 2 (in a different order). First of all (see Table 2, main learning 1), an undisputed aim facilitates the easy identification of opportunities for participation for all involved. We hypothesize that the Food Boost Challenge approach could also be applied to promote other challenging healthy eating behavior, such as more sustainable diets or drinking water. It would be interesting to assess if the approach could also be successfully used for achieving goals for other lifestyle and/or health-related aims or even non-health-related challenges.

Although large numbers of students participated within their curricular activities in the research phase (see Table 2, main learning 2), the numbers intended for the challenge phase were not met. Student teams participating in this phase took part on a voluntary, extracurricular basis. In our experience, and within the Dutch school system, a curricular nature of activities enables participation of large numbers of students, which also facilitates an equitable approach. However, this may differ from country to country [23]. For future editions, the focused efforts made for phase 1—to find smart combinations which allow students to participate in the challenge from within their curriculum—should be extended to the challenge phase (see Table 2, main learning 3).

With only 3 out of 25 ideas of insufficient quality for participation in the challenge, we consider the recruitment of student teams and information on the challenge to be adequate [19]. However, for accelerating innovation, we would recommend incorporating active dissemination of results of the research phase when recruiting students for the challenge phase (see Table 2, main learning 6), even if these results are preliminary. Apart from the events during which research results are shared, other innovative ways of sharing research findings with all involved should be sought. Continuous sharing of bite-sized findings, for example through micro-learning, might be a promising route [24] resulting in even more innovative ideas, concepts, and prototypes. Since the target group is highly active on social media, active sharing of findings through these media should also be explored.

Ultimately, the Food Boost Challenge is designed as an intervention which should increase adolescents’ intake of vegetable and fruit products. Although we conclude that the Food Boost Challenge generated a buzz, the effectiveness on intake and the wider impact has not yet been assessed. Additional funding and research are required to evaluate its impact using a relevant framework, such as RE-AIM [22] (see Table 2, main learning 5). In addition, the quantitative effect should be assessed. In order to minimize the research burden on adolescents, within this edition of the Food Boost Challenge, a number of students developed a quick quantitative scan for assessing adolescents’ consumption of vegetable and fruit products. However, development of these tools was hampered because too many adolescents in our population do not yet possess sufficient food literacy skills to accurately and precisely recognize portion sizes of vegetable and fruit products. Even the often recommended use of food photos did not sufficiently improve the accuracy of the estimates. Ironically, this underlines the relevance of increasing the knowledge and skills of this target group.

Not only participation of adolescents in the Food Boost Challenge has the potential to affect their attitude and/or intake of vegetables and fruit. Of course, the developed prototypes are also geared towards this aim. The expert jury concluded in the finals that this potential was highest for the prototype of Team Veggie Smooth. This finding is supported by a number of student research projects conducted in phase 1. Smoothies are a very popular product for this age groups, which has a healthy image for them. Although a source of liquid calories, this is mitigated by the high percentage of vegetables in this product (60%) and also by offering smaller portions (one sachet contains two servings). The public vote was won by Team Power Tower, a prototype which provides easily accessible vegetable snacks in between meals. In The Netherlands, vegetables are not often eaten as snacks, in between meals [16]. Introducing a new moment of use is a well-known marketing strategy to prevent cannibalism and increase consumption. This is particularly relevant for a bulky product, like vegetables, of which consumption during single occasions is often self-limiting. Team Veggie Smooth also won the prize to pitch their prototype for overseas investors. Follow-up investment in this prototype would fuel implementation of a potentially popular product, thereby increasing the chance that adolescents would actually increase their consumption of vegetables and fruit. Although these teams did not win a prize, partners which cater for schools and teachers and boards of pre-vocational schools showed great interest in implementing other prototypes, such as Eat 5 High 5, the Good Food Mood App, and Seasonal Food. As stated previously, there is no hard evidence of enhanced consumption yet; however, entirely in the spirit of participatory action research, these discoveries offer a promising start.

A final conclusion drawn from this first Food Boost Challenge is that this approach was a powerful way of creating a community of a wide variety of partners (see Table 2, main learning 4). This is a very good foundation for innovative practice-based research with impact in living labs. However, for achieving actual change in society, we would recommend adding a fifth phase, dedicated to implementation of high-potential prototypes, to the Food Boost Challenge. The exact implications for phase 1–4 and requirements for such a phase 5 should be developed in future editions of the Food Boost Challenge. While most partners in the current edition were satisfied, an implementation phase would provide added value for some of them. With dedicated partner management, these and other needs of partners could be identified and met [25] (see Table 2, main learning 4).

In the introduction, we refer to the WHO’s aim to reduce NCDs through a combination of overall principles [6], namely (1) life course approach; (2) equity-based approach; (3) multisectoral action; (4) empowerment of people and communities; (5) evidence-based strategies. We conclude that the design of the Food Boost Challenge as described in this paper contributes to these aims and could, therefore, contribute towards reducing NCDs. In the Food Boost Challenge, we focused on an often-overlooked target group, namely adolescents with lower education levels and students which we reached intra-curricularly (principles 1 and 2). A diverse array of partners participated in the Food Boost Challenge, ranging from NGOs to start-ups, scale-ups, and multinationals [19] (principle 3). During the cocreation phase, consortia were formed, with each consortium comprising a student team, representatives from partners, and members of the target group. Each initial consortium meeting was facilitated by a member of the core team. Consortia were empowered through professional training and actively opening up networks for them. Quite a few consortia have advanced plans for continuing the development of their innovative concepts to increase vegetable and fruit consumption in adolescents, some of them with their initial consortium partners and some with new business partners. Therefore, we conclude people have been empowered and communities have been strengthened (principle 4). Another example is that some of the activities during which students collaborated with schools in participatory research projects will be continued next academic year, with new generations of students and scholars. For future editions, effectiveness should be monitored to grow the evidence base of the Food Boost Challenge approach as an intervention which enhances the vegetable and fruit intake of adolescents (principle 5). 

## 5. Conclusions

In conclusion, the Food Boost Challenge is a four-phased approach which combines (a) direct engagement with adolescents; with (b) empowering them and students—also through collaboration with partners throughout the food system; with (c) the advancement of knowledge and insights into adolescents by student research in order to achieve—also through the generation of validated prototypes—(d) transformative action for enhancing the consumption of vegetables and fruit among adolescents. Therefore, we conclude the Food Boost Challenge is a participatory action research approach which shows potential to be adapted to promote various healthy eating behaviors among school students.

## Figures and Tables

**Table 1:** Brief description of the four phases of the Food Boost Challenge, as planned and executed, with details for each phase with regard to the main activities, goals, duration, main participants, and key output.

Element of Description	Preparation and Development of Food Boost Challenge	Phase 1: Research	Phase 2: Concept Challenge	Phase 3: Cocreation Prototypes	Phase 4: National Finals
Main activities	Recruitment of partners and funding; Development of design of Food Boost Challenge	Execution of student research, including data collection, analysis, presentation of results	Student teams challenged to submit innovative ideas	Matchmaking, prototype cocreation, including pitch event	National platform for teams and prototypes
Main goal	30 partners	100 students	50 student teams and ideas	15 consortia	100 visitors to final event
Duration ^1^	Start of partner recruitment until launch: 4 months; Launch until start of phase 1: 2 months; End of recruitment of partners: at start of Phase 3.	Student research projects varied in length from 4–10 months.	Challenge open for submission of concept ideas: 6 weeks.	Matchmaking: 1 day; Prototype cocreation in consortia: 2 months; Pitch event: 1 day.	Public voting: 1 week; Jury voting and national event: 1 day.
Main participants	Core team (n = 7)	200 students studying >1000 scholars; >30 teachers at research partners’ institutions and pre-vocational schools; 34 partners [19]; Core team complemented with 1 internship student and 1 project management support member	Open to all Dutch pre-vocational schools and universities (BSc and MSc level): 25 applications; Jury consisting of 3 core team members	10 finalist student teams; 15 adolescents representing the target group during cocreation; Partners throughout adolescent’s ecosystem [19]: ○ 4 schools; ○ 9 multinationals; ○ 5 start-ups and scale-ups; ○ 1 serious gaming industry partner; ○ 3 catering partners; ○ 4 connectors of social advocacy partners; ○ 3 governmental organizations; ○ 4 non-governmental organizations. Core team 3 external trainers	10 finalist student teams; 34 partners; 7 expert jury members; core team; wider audience (professionals, students, and target group); >1500 general public voters
Key output	Introductory video of official launch of Food Boost Challenge [20]; Slide deck for partner recruitment; Website [19].	Explanatory slide deck for partner kick-off and one for student projects; Generation and collection of research questions for student projects; Available for all partners and student teams: student project reports (according to their respective curricular requirements); Slide deck summarizing initial results.	Flyer enabling national recruitment of student teams; 25 innovative concepts in 4 directions; Brochure summarizing research results for consortia; 2 newsletters [19].	Matchmaking event consisting of 10 live pitches; 4 rounds of speed date sessions to match student teams and partners and target group into consortia for cocreation; Targeted cocreation training; validation and prototyping projects by consortia; Hands-on pitch training; 10 video-pitches [20]; 3 newsletters [19].	Public voting based on 10 video-pitches; National finals existing of experiencing prototypes by jury and audience; 10 live pitches (2 min and 5 min questions); Award ceremony; Slide deck summarizing research findings; Networking opportunity for all present at final event; 1 after-movie [20]; 90-s animated explainer about the Food Boost Challenge [21].

^1^ See Figure 1 for a graphical representation of the timeline of the Food Boost Challenge.

**Table 2:** Goals, realization of goals and learnings of the Food Boost Challenge.

Goals	Realization of Goals	Learnings and Key Drivers of Success
>100 students and scholars focus on healthy eating behavior by participation in student research projects	Projects of students (>200 from 8 departments at The Hague University of Applied Sciences) interactively collected data of >1000 pre-vocational scholars in and near The Hague	Goal exceeded The focus on the Food Boost Challenge’s undisputed aim (i.e., enhancing adolescent’s intake of vegetable and fruit products) enabled easy identification of opportunities for teachers at schools and universities (main learning 1); The flexibility of approach for projects, as long as they contributed towards the above aim, enabled collaborating through intra-curricular projects; The intra-curricular nature of projects, both at universities and at schools, enabled participation of large numbers of students and scholars (main learning 2); Dedicated project leader required to mix and match research questions, school, and university needs; Dedicated contact person required at participating schools for further distribution of projects; Regular meetings between contact persons and project leader required.
>50 fresh and healthy ideas	25 ideas	Goal not reached Reasons unknown: ○ More time might be required for recruiting student teams, especially during a strict COVID-lockdown; ○ A more curricular nature of activities might have enabled participation of a larger numbers of students (main learning 3, see also main learning 2 above) All but 3 ideas were of sufficient quality to be eligible for selection in the prototyping phase.
15 selected ambitious and complimentary teams	10 teams	Goal modified during challenge phase Due to a limited number of applications (25 instead of the expected 50) and budget constraints (solely funding by partners with no additional grants obtained), there was a reduction in the number of teams selected for the cocreation phase. Teams from 10 different universities in The Netherlands (BSc and MSc level).
2 awards (public and expert jury)	4 awards + testing facilities	Goal exceeded In addition to the 2 monetary prices, 1 partner provided a non-monetary price and 1 an opportunity to pitch on their overseas location for their American investors; One organization offered student teams testing facilities at the Floriade.
30 winning partners	34 partners [19]	Goal exceeded Cash contributions—or funding—are essential for professional training, events, and project management support; In-kind contributions by partners in consortia were invaluable for student teams; student insights were invaluable for partners; See Table 1, phase 3 for diverse array of partners. The participation of each partner is differently motivated. In order to satisfy their needs, dedicated partner management was required (main learning 4).
National buzz and boost for healthy eating behaviors of pre-vocational adolescents	Large exposure among adolescents via participating scholars and students, among their teachers and colleagues, in the professional network of partners and core team members; and among the general public	Goal reached “Buzz” measured by exposure: ○ Student teams (of 10 different universities throughout The Netherlands) tested their concepts and prototypes with >1000 pre-vocational scholars throughout the Netherlands; ○ >2000 members of the general public viewed the video-pitches about the prototypes promoting healthy eating behaviors of school students; ○ >1500 members of the general public voted for one of the ideas. In future editions: ○ “Boost” should be studied qualitatively using the RE-AIM framework [22] and quantitatively using relevant surveys (main learning 5); ○ Incorporating active dissemination of results of the research phase when recruiting students for the concept challenge and throughout the phase of cocreation of prototypes is recommended (main learning 6).
Start of a national movement	Next edition of the Food Boost Challenge started in a different region in The Netherlands [19]; Advanced plans, including submitted grant application, for future editions of the Food Boost Challenge both in the Netherlands and abroad; Submitted grant application for design-based research into a challenge-based learning concept where the Food Boost Challenge is one of the pilot cases; Several consortia continued development in order to realize their concepts.	Goal exceeded The community of Food Boost Challengers is growing (main learning 4).

**Table 3:** Prototypes presented at the finals.

Name of Student Team	Tagline of Prototype	Brief Description of Prototype	Innovation Route(s) of Prototype
Fused Food	The healthiest veggies in the tastiest meat	Burgers, sausages, and Dutch meatballs containing at least 30% plant-based material.	Product
Power Tower	Healthy snacks at your fingertips	Stackable cubes for the fridge door containing snack vegetables, such as mini cucumbers, snack tomatoes, and baby carrots.	Technology
Fruit to go	Trendy fruit bar in supermarkets	Fruit bar with freshly cut and assembled fruits in your local supermarket.	Route to market
Good Food Mood App	Fruit and vegetables for mental health	One-stop app for adolescents informing them about vegetables and fruit and mental health and well-being, plus a social support network.	Technology
Eat 5 High 5	CakeBiteFavorites, your favorite way to eat healthy	Promotion of vegetable and fruit intake for adolescents through a three-pillared approach: (1) healthy cakes containing fruits or vegetables; (2) organizing social events and networking via social media; (3) an app for sharing healthy recipes, tracking of vegetable and fruit intake (eat 5), and sharing achievements (high 5).	Product and technology
Tasty tin	The vending machine concept of future health	A vending machine containing healthy vegetable- and fruit-rich snack products packaged in recyclable tins, designed to become collector’s items. Machines will be coupled to an app providing personal offers and challenges for users.	Route to market and technology
Veggie Smooth	Veggies and fruit disguised as healthy smoothies	Packages containing frozen fruit (40%) and vegetables (60%) from local residual products in combinations that match adolescent’s taste. A package contains two servings and can be prepared in a regular blender after adding water.	Product and route to market
Veggie Cookie	Take a break to get your veggie intake	Cookies containing vegetables, fruit, and legumes.	Product
Vendy	We put the vegetable in your candy	A sustainably made, healthy and desirable sharable bar containing at least 50% vegetables and fruit.	Product
Seasonal food	Seasonal food tastes good. Adolescents love to cook this food.	Seasonal vegetable and fruit challenges for adolescents at high schools. Each season, adolescents will be challenged to make their favorite fruit- or vegetable-rich recipes. They will be attracted by the prospect of prizes and awards. Other adolescents can buy the seasonal products and will receive the recipes.	Hotspot

## Data Availability

The data collected in the student research projects presented in this study are available on request from the corresponding author. The data are not publicly available due to the fact that they are made as part of their studies, i.e., a learning process. The student teams’ ideas presented in this study are openly available and can be found here https://www.youtube.com/@FoodBoostChallenge/videos, accessed on 17 May 2022.
